# Inhibitory Effects of Palmatine on P2X7 Receptor Expression in Trigeminal Ganglion and Facial Pain in Trigeminal Neuralgia Rats

**DOI:** 10.3389/fncel.2021.672022

**Published:** 2021-07-22

**Authors:** Cancan Yin, Wenhao Shen, Mingming Zhang, Lequan Wen, Ruoyu Huang, Mengyun Sun, Yun Gao, Wei Xiong

**Affiliations:** ^1^Affiliated Stomatological Hospital of Nanchang University, Nanchang, China; ^2^Hangzhou Stomatology Hospital, Hangzhou, China; ^3^Department of Physiology, Basic Medical College, Nanchang University, Nanchang, China; ^4^Joint Program of Nanchang University and Queen Mary University of London, Nanchang, China; ^5^Jiangxi Provincial Key Laboratory of Autonomic Nervous Function and Disease, Nanchang, China; ^6^Jiangxi Provincial Key Laboratory of Oral Biomedicine, Nanchang, China

**Keywords:** trigeminal neuralgia, palmatine, P2X7 receptor, trigeminal ganglion, chronic constriction injury of the infraorbital nerve

## Abstract

Trigeminal Neuralgia (TN) refers to recurrent severe paroxysmal pain in the distribution area of the trigeminal nerve, which seriously affects the quality of life of patients. This research applied the chronic constriction injury of the infraorbital nerve (CCI—ION) approach to induce an animal model of TN in rats. The mechanical pain threshold of each group of rats was determined postoperatively; the expression of P2X7 receptor in trigeminal ganglion (TG) was assessed by qRT-PCR, immunofluorescence and Western blot; and the changes of the proinflammatory cytokines IL-1β and TNF-α in serum of rats were detected by ELISA. The results showed that the administration of palmatine in the TN rats could reduce the mechanical pain threshold, significantly decrease the expression of P2X7 receptor in TG, and lower the serum concentrations of IL-1β and TNF-α, compared to the sham group. In addition, the phosphorylation level of p38 in TG of TN rats was significantly decreased after treatment with palmatine. Likewise, inhibition of P2X7 expression by shRNA treatment could effectively counteract the adversary changes of pain sensitivity, IL-1β and TNF-α production, and p38 phosphorylation in TN rats. Our data suggest that palmatine may alleviate mechanical facial pain in TN rats possibly by reducing the expression of P2X7 receptor in TG of TN rats, which may be attributable to inhibiting p38 phosphorylation and reducing the release of IL-1β and TNF-α.

## Introduction

Trigeminal Neuralgia (TN) is a severe, recurrent pain that occurs in the distribution area of the trigeminal nerve, displaying sufferings such as knife cutting, acupuncture, electric shock or burning; its frequency and pain intensity increase with the duration of the disease (Wu et al., 2019). It occurs most often in the second and/or third trigeminal branches, with the right side affected slightly more frequently than the left (Maarbjerg et al., 2014). Its frequency is estimated to be 12.6–28.9 per 100,000 persons per year in the general population (van Hecke et al., 2014), seriously affecting the quality of life of patients. At present, the management methods for TN include drug therapy and surgical treatment, but there are certain limitations and deficiencies in either treatment method (Nova et al., 2020). Therefore, it is of great significance to explore new molecular targets for the treatment of TN.

P2X receptor is a ligand gated ion channel activated by extracellular ATP (Leng et al., 2019), and contains P2 × 1–7 subtypes (Tang et al., 2018). The P2X7 receptor is activated by relatively high ATP concentrations (Ursu et al., 2014). ATP activates microglia cells through P2X7 receptor under pathological conditions, leading to increased release of cytokines, including IL-1β, IL-6, IL-10, TNF-α etc. (Li et al., 2017). Chessell et al. (2005) found that P2X7 receptor expression was increased in dorsal root ganglion and injured nerves in patients with chronic neuropathic pain. In addition, we also observed that P2X7 receptor expression in TG was increased in rats of TN model established by the chronic constriction injury of the infraorbital nerve (CCI—ION) (Xiong et al., 2019). Overexpression of the lncRNA uc.48 + by designed uc.48 + plasmid transfection has been verified to be capable of augmenting P2X7 receptor expression in TG, and then might be responsible for the anomaly excitable signal transduction and pain transmission in TN (Xiong et al., 2019). Therefore, the study on the role of P2X7 receptor in TN may offer a new direction for the treatment of TN.

Palmatine is a proberberine alkaloid extracted from the procellulose of the root cortex used in Chinese traditional medicine (Jung et al., 2014). It exhibits anti-inflammatory and antibacterial effects (Küpeli et al., 2002), mainly used for treatments against gynecological inflammation, respiratory tract infection, urinary tract infection, bacterial dysentery, enteritis, and conjunctivitis (Chao et al., 2009; Pathan et al., 2015). The aim of this study was to investigate the effect of palmatine on P2X7 receptor in TG of TN rats.

## Materials and Methods

### Animals

Healthy adult male Sprague-Dawley (SD) rats weighing 180–200 g were provided by the department of animal science, Jiangxi University of Traditional Chinese Medicine. Our experiments followed the international association for pain research (IASP) ethical guidelines for animal pain research. TN rat model was established by chronic constriction injury of the infraorbital nerve (CCI-ION) approach as detailed below (Xiong et al., 2017, 2019).

### Chronic Constriction Injury of the Infraorbital Nerve (CCI-ION)

The rats were anesthetized by intraperitoneal injection of chloral hydrate (Shanghai Maclin Biochemical Technology Co., Ltd., batch no.: C10232286, China). After skin preparation and disinfection, a curved incision was made on the eyebrows and blunt anatomical separation was performed with a glass minute needle; the skull, frontal bone and nasal bone were visible until the eye socket appeared. At this point, the contents of the orbit were pushed aside with the glass needle to expose the inferior orbital nerve at the base of the medial orbit. The suborbital nerve was loosely ligated by microsurgical technique (5–0 chromic gut) for 2 ligatures. The interval between the two knots is about 1 mm. All CCI-ION rats were carried out by the same researcher to avoid technical differences. The criterion for suborbital nerve compression is a slight narrowing of the diameter of the nerve fibers without affecting nerve transmission and blood circulation. Finally, conventional incision suture and disinfection were performed, and the rats were fed and given access to water normally after waking. In the sham rats, there was no nerve damage.

### Experimental Design

Healthy adult male SD rats were randomly divided into six groups: Sham control group, sham + palmatine treatment group, TN model group, TN + palmatine treatment group, TN + P2X7 shRNA treatment group and TN + scramble shRNA treatment group. In the Sham group, the inferior orbital nerve was exposed without ligation. Rats in the sham + palmatine group and TN + palmatine group were given intraperitoneal injection of palmatine (0.02 ml/100 g) on the first day after surgery and were injected continuously for 14 days. Rats in the TN + P2X7 shRNA group and TN + scramble shRNA group received P2X7 shRNA and scramble shRNA by intravenous injections, respectively, on the 10th day after surgery.

### Measurement of the Mechanical Withdrawal Threshold (MWT)

Noxious-pressure stimulation was used to evaluate mechanical hyperalgesia.

Unrestrained rats were placed in a clear plastic chamber (22 × 12 × 22 cm) on a stainless-steel mesh floor and allowed to acclimate. The mechanical pain sensitivity threshold of the operative side of each rat was measured by an electronic painometer (BME-404 NO. E5489, Inst. of BME, CAMS&PUMC), and the tentacles on the operative side of the nasal area and its surrounding hairy skin were stimulated regularly 1 day before and 1, 3, 5, 7, 11, 13, and 15 days after the operation. The rats were stimulated when they sniffed or were inactive, i.e., Four paws on the ground, neither moving nor frozen, but showing sniffing behavior. The main positive reactions of the rats were: (1) quick dodge, back or turn, etc.; (2) aggressive behaviors: Fast scratching and biting of stimuli; (3) scratching the face, occurring at least 3 consecutive scratches on the stimulating part. Each rat was stimulated on the surgical side six times, at 1 min intervals. All rats underwent adaptive training of mechanical stimulation 1 week before operation.

### Quantitative Real-Time PCR

On the 15th day after the operation, the rats in each group were given 10% chloral hydrate intraperitoneal injection for anesthesia. After decapitation, the TGs in the operative side were removed. Total RNA was extracted according to the instructions of the RNA extraction kit (TransGen, Beijing, China). The cDNA synthesis was performed with a TransScript First Strand cDNA Synthesis Super Mix (TransGen, Beijing, China) stored in the refrigerator at –20°C.

The sequences of primers are as follows:for β-actin, forward 5′- AAGATCCTGACCGAGCGTGG and reverse 5′- CAGCACT GTGTTGGCA TAGAGG; for P2X7, forward 5′- AGCGTGAA TTACGGCACCAT and reverse 5′-CAAAGGGAGGGTGTAGT CGG; for IL-1β, forward 5′- CCTATGTCTTGCCCGT GGAG and reverse 5′-ACTGCTGGACGATCACACAC; and for TNF-α, forward 5′- CACGTCGTAGCAAACCACCAA and reverse 5′-T ACCTAGAGTTTCTGTT GGTTG.

Quantitative real-time PCR (qRT-PCR) was performed using the SYBR^®^ Green Master Mix on an ABI PRISM^®^ 7500 Sequence Detection System (Applied Biosystems Inc., Foster City, CA). The specificity of amplification was determined according to the dissolution curve, and the mRNA level in TG of rats in the corresponding group was calculated according to the Ct value with β-actin as the internal reference.

### Immunofluorescence

On the 15th day after the operation, rats in each group were intraperitoneally injected with 10% chloral hydrate for anesthesia, and TGs were perfused with 4% PFA. After dehydration in 30% sucrose solution (changed every 8 h) for 24 h, the dehydrated tissue was embedded and solidified in embedding solution and cut into 8 μm thick sections using a –20°C frozen slicer (Leica). The sections were rinsed with PBS and then dripped with 0.3% Triton x-100. After being placed at room temperature for 15 min, the sections were blocked with goat serum in a water bath at 37°C for 1 h. The sections were then incubated with diluted (1:100 in PBS) primary antibodies against glial fibrillary acidic protein (GFAP) (chicken anti-GFAP, Abcam) and P2X7 (rabbit anti-P2X7, Abcam) overnight at 4°C. After washing with PBS, sections were incubated with diluted (1:200 in PBS) secondary antibody [goat anti-rabbit TRITC (tetraethyl rhodamine isothiocyanate; Jackson ImmunoResearch Inc., West Grove, PA, United States) and goat anti-chicken FITC (Beijing Zhongshan Biotech Co.)] in a 37°C-water bath for 1 h. After washing with PBS, the nuclei were stained with 0.1% DAPI for 10 min. In this part of the experiment, there were 6 animals in each group. One of every 5 consecutive slices of TG of each animal was selected, and at least 3 slices were immunofluorescence stained. To specify the immunoreactivity of P2X7 antibody, as a negative control, normal serum was substituted for the primary antibody. The background was determined by averaging the optical density of 10 random areas. The sections were imaged using a fluorescence microscope (Olympus, Tokyo, Japan). Results were analyzed using Image-Pro Plus6.0 software.

### Western Blotting

On the 15th day after the operation, the rats in each group were given 10% chloral hydrate intraperitoneally, and then their heads were cut off. The intraoperative TG was removed and washed with PBS. The tissues were homogenized in lysis buffer containing 10% protease inhibitors and 10% phosphatase inhibitors on ice. The homogenates were moved into 1.5 ml Eppendorf tubes and centrifuged at 4°C for 10 min at 12,000 rpm to obtain the supernatants. After adding 6x loading buffer, the supernatants were mixed well by vortex, put in boiling water for 5 min, and cooled at room temperature before being stored at –20°C. Samples containing equal amounts of protein (20 μg) were separated by SDS-polyacrylamide gel (12%) electrophoresis with a Bio- Rad system. Following electrophoretic transfer onto PVDF membrane using the same system, the PVDF membrane was blocked for 2 h in 5% skim milk prepared with 1 × TBST solution. The membranes were incubated with one of primary antisera (β-actin, Beijing Zhongshan Biotech. Co., Beijing, China; rabbit anti-P2X7 1:500, Abcam, England; rabbit polyclonal anti-p38 and rabbit polyclonal anti-P-p38 1:500, Cell Signaling, United States) in the same buffer overnight at 4°C. PVDF membrane was washed with 1 × TBST solution and incubated with secondary antibody (horseradish peroxidase conjugated goat anti-rabbit IgG, 1:2,000; Beijing Zhongshan Biotech. Co., Beijing, China) on ice for 2 h. After washing, the membrane was washed with 1 × TBST solution, and incubated with the chemiluminescence solution (batch no.: S6009; Suzhou Yuhengwu Technology Co., Ltd., China) to develop the protein bands in a dark chamber of the exposure machine (Bio-Rad, United States). The optical density of protein bands was analyzed by image-pro Plus Image analysis software, and the optical density of target proteins (P2X7, p38, and P-p38) in each group was normalized to the β-actin bands in their corresponding groups to determine the expression levels.

### Enzyme Linked Immunosorbent Assay (ELISA)

On the 15th day after the operation, the rats in each group were given 10% chloral hydrate intraperitoneally and decapacitated immediately after anesthesia. Fresh blood was collected and put into the centrifuge tube at room temperature. Serum was extracted after centrifugation at 1,200 rpm for 15 min. The levels of IL-1β and TNF-α in each sample were assessed using an ELISA kit (Wuhan Shenke, Wuhan, Hubei, China) according to the instructions of the manufacturer. In brief, a sequence of 5 standard solution was prepared by consecutive dilutions from 30x stock. Then 50 μl standard or dilution buffer (blank) was added into a well in the 96-well plate coated with enzyme-labeled antibody. For test samples, 40 μl diluent buffer and 10 μl serum was added into each well. All tests were in duplicate. After sealed by a covering membrane, the plate was shaken gently to mix the solution. The plate was placed in 37°C water bath for 30 min, followed by discarding the liquid. Then washing liquid was added into each well, followed by leaving plate to stand for 30 s and discarding the washing solution; this washing process was repeated 5 times. Afterward, 50 μl enzyme reaction reagent was added to each well including the blank. The plate was sealed by covering membrane again and incubated in a 37°C-water bath for 30 min. Then, the liquid was discarded, and the plate was washed 5 times as described above. Then 50 μl color developing agent A and 50 μl color developing agent B were added into each well. After gently shaking and mixing, the place was placed in 37°C water and bathed in the dark for 10 min. Finally, 50 μl termination solution was added to each hole to stop the reaction. The absorbance expressed as optical density (OD value) of each well was measured by a multifunctional microplate reader at the wavelength of 450 nm. A standard curve was drawn based on the OD value according to the kit instructions. The concentrations of IL-1β and TNF-α in serum samples were calculated using the OD value of each sample against the standard curve after correction of dilution factors.

### Statistical Analysis

All results were expressed as the mean ± SEM, and statistical analyses were performed using SPSS 21.0 software. Differences between treatment groups were analyzed by ANOVA followed by Dunnett’s *post hoc* test for multiple comparisons. *P*-values < 0.05 were considered to be statistically significant.

## Results

### Effect of Palmatine on Mechanical Pain Threshold of TN Rats

**FIGURE 1:**
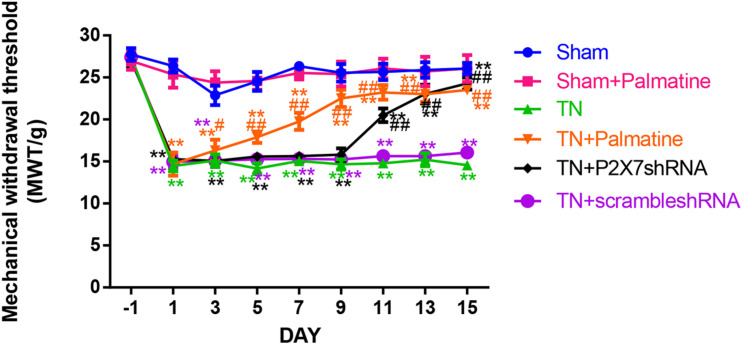
The change of the mechanical withdrawal threshold (MWT) of rats in each group. Values are mean ± SD, *n* = 6, **p < 0.01 vs. sham group, ^#^*p* < 0.05 and ^##^*p* < 0.01 vs. TN group.

Before surgery, there was no significant difference in the mechanical pain threshold of the lateral sensory area between groups (*p* > 0.05). 15 days after surgery, the mechanical pain sensitivity threshold of the lateral sensory region of the TN group was significantly decreased compared with the Sham group (*p* < 0.01). After palmatine or P2X7 shRNA treatment, the mechanical pain threshold of the lateral sensory area in the TN + palmatine group and TN + P2X7 shRNA group was significantly higher than that in the TN group (*p* < 0.01) (Figure 1).

### Effect of Palmatine on mRNA Levels of P2X7 Receptor, IL-1β, and TNF-α in TGs by qRT-PCR

**FIGURE 2:**
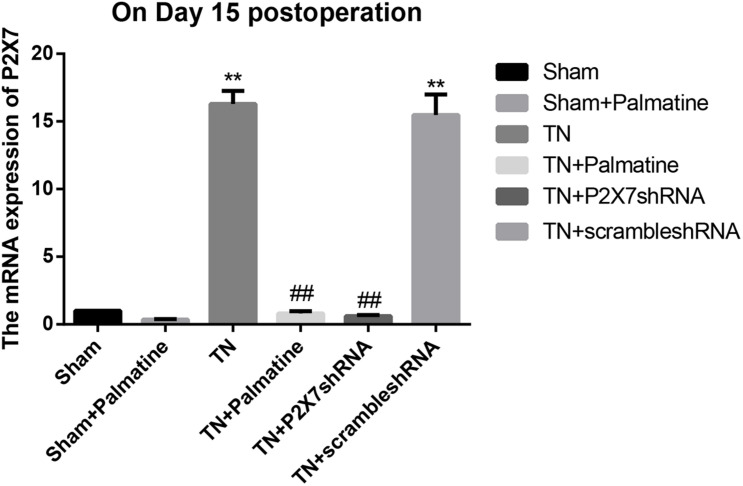
P2X7 mRNA expression in TGs of each group detected by qRT-PCR on 15 days after operation. Values are mean ± SD, *n* = 6, **p < 0.01 vs. sham group,^##^*p* < 0.01 vs. TN group.

**FIGURE 3:**
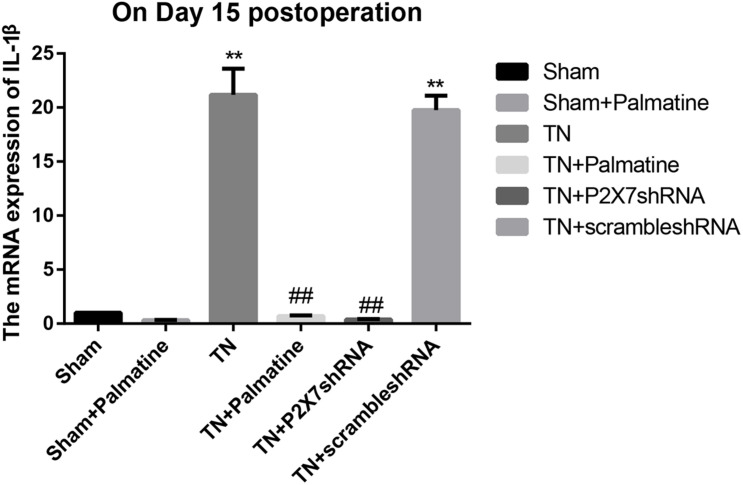
IL-1β mRNA expression in TGs of each group detected by qRT-PCR on fifteen days after operation. Values are mean ± SD, *n* = 6, **p < 0.01 vs. sham group, ^##^*p* < 0.01 vs. TN group.

**FIGURE 4:**
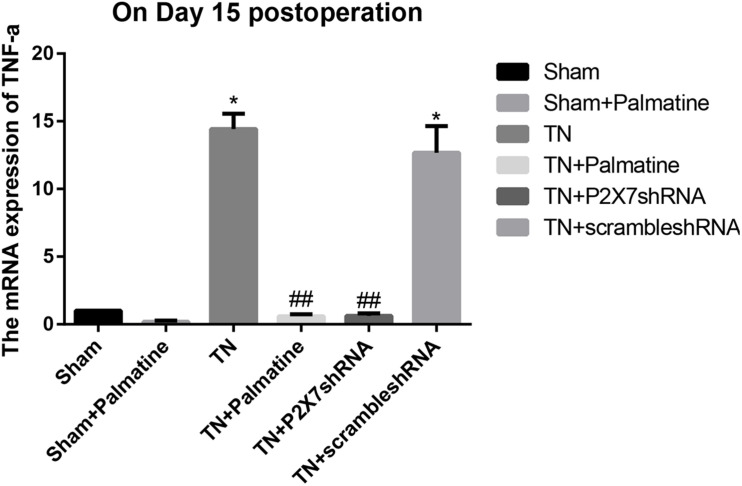
TNF-α mRNA expression in TGs of each group detected by qRT-PCR on 15 days after operation. Values are mean ± SD, *n* = 6, *p < 0.05 vs. sham group, ^##^*p* < 0.01 vs. TN group.

The mRNA levels of P2X7, IL-1β, and TNF-α in intraoperative TGs in rats of each group was detected by qRT-PCR 15 days after surgery. The results showed that the mRNA levels of P2X7, IL-1β, and TNF-α were significantly elevated by 15–16, 19–21, and 13–15-folds in the TN group compared with the sham group (*p* < 0.05). After palmatine or P2X7 shRNA treatment, the mRNA levels of P2X7, IL-1β, and TNF-α in intraoperatively TGs of the TN + palmatine group and TN + P2X7 shRNA group were significantly decreased compared with those of the TN group (*p* < 0.01) (Figures 2–4).

### Effect of Palmatine on the Expression of P2X7 Receptor in TGs by Immunofluorescence

**FIGURE 5:**
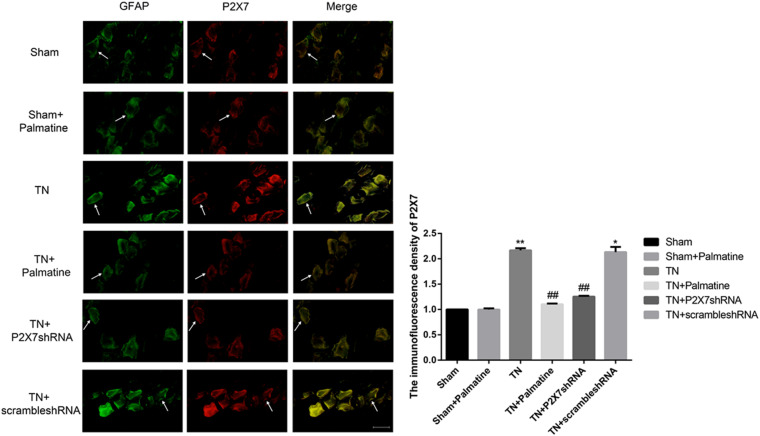
The co-expression of P2X7 and GFAP in the intraoperative TGs of rats in each group was detected by immunofluorescence. The arrows point to glial cells that are immune-stained. Scale bar: 100 μm. Values are mean ± SD, *n* = 6, *p < 0.05 and **p < 0.01 vs. sham group, ^##^*p* < 0.01 vs. TN group.

At 15 days after surgery, P2X7 receptor expression in intraoperative TGs in rats of each group was also examined by double-labeling immunofluorescence. Compared with the Sham group, the expression of P2X7 receptor in the TN group was significantly increased (*p* < 0.01). After palmatine or P2X7 shRNA treatment, P2X7 receptor expression of TGs in the TN + palmatine group and TN + P2X7 shRNA group was significantly reduced compared with that in the TN group (*p* < 0.01) (Figure 5).

### Effects of Palmatine on the Phosphorylation of p38 in TGs

**FIGURE 6:**
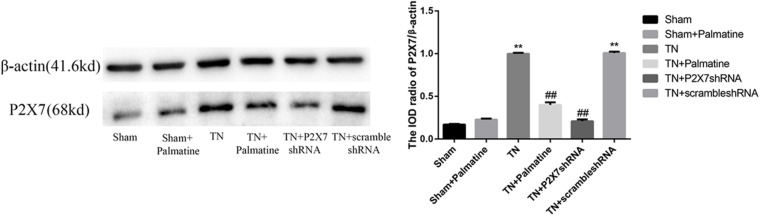
Effects of palmatine on the expression of the P2X7 receptor in TGs of each group on day 15 postoperation assessed by Western blotting. Values are mean ± SD, *n* = 6, **p < 0.01 vs. sham group, ^##^*p* < 0.01 vs. TN group.

**FIGURE 7:**
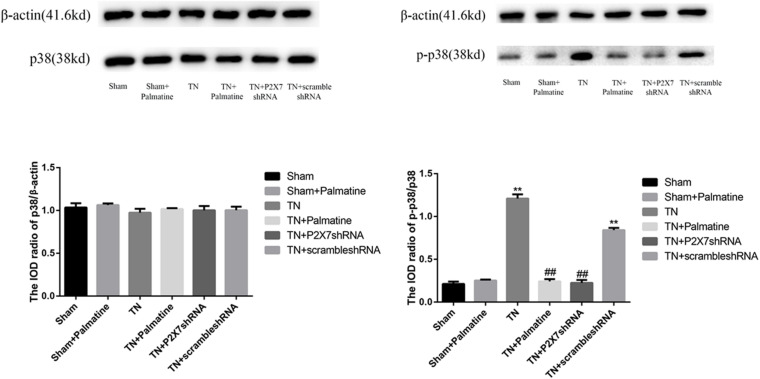
Effects of palmatine on the phosphorylation of p38 in TGs detected by Western blotting. Using image analysis, the integrated OD values of P-p38 were normalized to p38/β-actin. Values are mean ± SD, *n* = 6, **p < 0.01 vs. sham group, ^##^*p* < 0.01 vs. TN group.

The expression level of P2X7 receptor and the phosphorylation level of p38 in TGs were also studied by Western blotting. Similar to the observations from qRT-PCR and immunofluorescence, P2X7 receptor expression at the protein level was significantly increased in TN group compared with Sham group (*p* < 0.01). However, after palmatine or P2X7 shRNA treatment, P2X7 receptor content in intraoperatively TGs was significantly decreased in the TN + palmatine group and TN + P2X7 shRNA group compared with the TN group (*p* < 0.01) (Figure 6). There was no significant change in total p38 of TGs in each group (*p* > 0.05). By contrast, the expression of phosphorylated p38 (P-p38) in the TN group was significantly higher than that in the sham group (*p* < 0.01). But after palmatine or P2X7 shRNA treatment, P-p38 content was significantly decreased in the TN + palmatine group and TN + P2X7 shRNA group (*p* < 0.01) (Figure 7). These results indicate that the treatment of palmatine can effectively reduce the phosphorylation level of p38 in TGs of TN rats.

### Effects of Palmatine on Serum Levels of IL-1β and TNF-α in TN Rats

**FIGURE 8:**
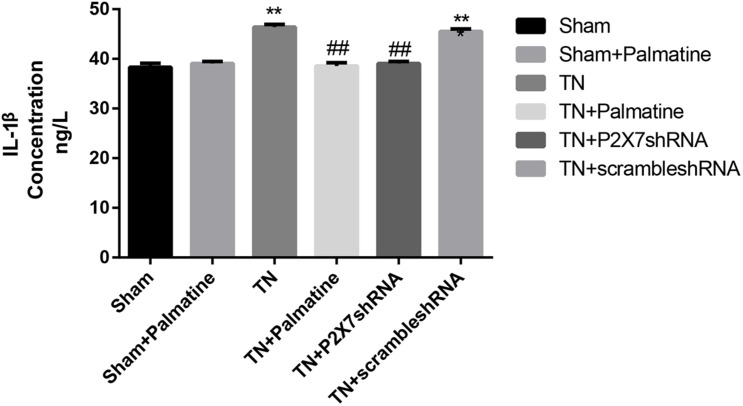
The concentrations of IL-1β in serum of each group rats determined by ELISA. Values are mean ± SD, *n* = 6, **p < 0.01 vs. sham group, ^##^*p* < 0.01 vs. TN group.

**FIGURE 9:**
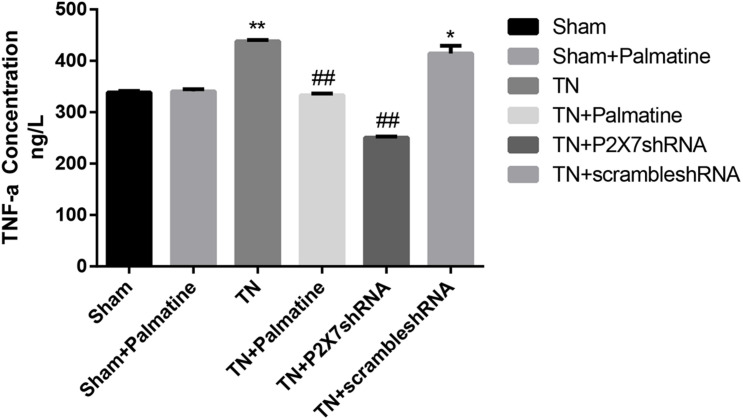
The concentrations of TNF-α in serum of each group rats determined by ELISA. Values are mean ± SD, *n* = 6, *p < 0.05 and **p < 0.01 vs. sham group, ^##^*p* < 0.01 vs. TN group.

Serum concentrations of the pro-inflammatory cytokines IL-1β and TNF-α in rats of each group 15 days after surgery were detected by ELISA. The results showed that the serum IL-1β and TNF-α were increased in TN group compared with sham group (*p* < 0.05). After palmatine or P2X7 shRNA treatment, serum IL-1β and TNF-α concentrations in the TN + palmatine group and TN + P2X7 shRNA group were significantly decreased compared with that in the TN group (*p* < 0.01) (Figures 8, 9).

## Discussion

TN is a common neuropathic disorder in the maxillofacial region, which has seriously affected the quality of life of patients, and even attributed to suicidal feeling (Zakrzewska et al., 2017; Freeman et al., 2019). Depression is also more common in patients with TN (Obermann, 2019). In clinical practice, TN is usually divided into primary and secondary types. In this work, the primary TN was studied. At present, the main methods of treating TN are drug therapy and surgical operation; but both of them have some drawbacks. Carbamazepine is preferred for drug treatment. However, Stefano et al. (2014) found that in 100 patients with TN treated with carbamazepine, 27% of them produced undesirable side effects, resulting in treatment interruption or dose reduction to the unsatisfactory levels. Surgical treatment is chosen when medication fails. Surgical treatment includes microvascular decompression, radiofrequency thermocoagulation, and gamma-knife radiotherapy, etc. Microvascular decompression is considered as a classic and effective surgical method for the treatment of TN, but craniotomy may cause trauma, postoperative complications such as facial numbness, cerebrospinal fluid leakage, intracranial hemorrhage and deafness (Li et al., 2019b). Continuous radiofrequency thermocoagulation is also widely used in the clinical treatment of TN, which may produce a pain relief rate of more than 90% (Teixeira et al., 2006). However, postoperative complications such as facial numbness, numbness of forehead and hearing impairment may also occur due to radiofrequency temperature (Huang et al., 2014; Yao et al., 2016). Furthermore, Gamma-knife surgery is very expensive, which limits its widespread use (Obermann, 2019). Thus, it is necessary to search for new molecular targets for the prevention and treatment of TN.

P2X7 receptor is a purinergic receptor functioning as non-selective cation channel activated by extracellular ATP (Kambur et al., 2018). It is highly expressed in epithelial cells, immune cells (T cells, macrophages, mast cells, and microglia), oligodendrocytes of the central nervous system, and Schwann cells of the peripheral nervous system (Di Virgilio et al., 2018; Kaczmarek-Hajek et al., 2018). It plays a role in inflammation while immune signaling is one of the most well studied functions (Kopp et al., 2019). A growing number of studies have shown a close relationship between P2X7 receptors and chronic inflammation and neuropathic pain (Yang et al., 2015; Liu et al., 2016; Luchting et al., 2016; He et al., 2017; Yi et al., 2018). The research results of our laboratory (Xiong et al., 2019) also indicate that the expression of P2X7 receptor in TG is increased in TN rats, but its effect on TN and its specific transmission mechanism are still unclear. In this study, TN model was established by CCI-ION approach, and the postoperative mechanical pain sensitivity threshold of these TN rats was significantly decreased. The results of qRT-PCR, immunofluorescence and Western blotting showed that the expression of P2X7 receptor in TGs of TN rats was significantly upregulated. Immunofluorescence results confirmed that P2X7 receptor and GFAP (a specific marker for satellite glial cells) were co-expressed in TGs of TN rats, and such co-expression was stronger than that of sham rats. Therefore, our data suggest that P2X7 receptor on satellite glial cells of TGs is involved in the development of TN and may be an effective therapeutic target.

Palmatine has been widely used in clinical treatment for inflammation, dysentery, jaundice, hypertension and liver-related diseases (Zhang et al., 2012). In addition, studies (Hambright et al., 2015; Ishikawa et al., 2016; Chakravarthy et al., 2018; Johnson-Ajinwo et al., 2019) have shown that palmatine has certain therapeutic benefits for pancreatic cancer, osteoporosis, prostate cancer, ovarian cancer and colon cancer (Liu et al., 2020b). Our work revealed that 2 weeks after the treatment of TN rats with palmatine, the reduced mechanical pain threshold was significantly increased, indicating that palmatine could alleviate the neuropathic pain caused by TN. Previous work in our laboratory has shown that palmatine treatment can inhibit trigeminal neuralgia, which may be related to the down-regulation of the expression of BDNF and CGRP in trigeminal ganglion neurons (He et al., 2020; Liu et al., 2020a). Moreover, after treatment with palmatine or P2X7 shRNA, the upregulated P2X7 expression at both mRNA and protein levels in TN rats was significantly counteracted. This indicates that TN caused by CCI-ION approach can activate P2X7 receptor in TGs. Studies have shown that P2X7 receptor activation stimulates the production and release of IL-1β and TNF-α (Chessell et al., 2005), and our ELISA results are consistent with this notion, showing increased serum concentrations of inflammatory cytokines IL-1β and TNF-α in TN rats. In addition, palmatine or P2X7 shRNA treatment could also protect TN rats from the enhanced production of IL-1β and TNF-α. This study revealed that palmatine therapy can relieve TN by inhibiting the expression of P2X7 receptor in TGs via reducing the production of inflammatory cytokines.

Studies have shown that P2X7 receptor activation involves ERK signaling pathway (He et al., 2017), and activation of the microglia p38MAPK pathway, which might be the response to the increased microglial calcium ion concentration (Li et al., 2019a), leads to hypersensitivity to pain in rats when pressed on the trigeminal nerve root (Han et al., 2012; Jeon et al., 2012). In order to further explore the effect of palmatine on P2X7 receptor-mediated TN, we examined the classical MAPK pathway component P-p38 at the protein level. Our results showed that there was no significant difference in the expression of total p38 protein in TGs in each group, but the level of P-p38 protein in TN rats was significantly elevated. Again, palmatine treatment could significantly inhibit such increased P-p38 levels in TN rats, an effect similar to the treatment by P2X7 shRNA. These observations indicate that TN-caused activation of P2X7 receptor increasing phosphorylation of the p38 MAPK pathway may act as the mechanism for palmatine to mitigate facial pain in TN rats.

Based on the above experimental results and the previous experimental results in our laboratory, we speculated that palmatine treatment could inhibit the activation and expression of P2X7 receptor on the satellite glial cells of trigeminal ganglion, inhibit the phosphorylation of the p38 pathway, down-regulate the level of inflammatory factors, and affect the communication between neurons and glial cells. Inhibition of the communication between neurons and glial cells can down-regulate the expressions of BDNF and CGRP in trigeminal ganglion neurons, and ultimately inhibit the pain transmission of trigeminal neuralgia.

## Conclusion

In conclusion, increased expression of P2X7 receptor is involved in the transmission of facial pain in TN rats. Palmatine can reduce the expression of P2X7 receptor, and the underlying mechanism may include inhibiting the phosphorylation of p38 MAPK in TGs, suppressing the release of IL-1β and TNF-α, and consequently reducing the mechanical pain sensitivity in the face of TN rats. Due to the complexity of TN, multiple pathways and receptors are involved in the progression of TN, and the precise mechanism of interaction between palmatine and P2X7 receptor still needs further research in the future.

## Data Availability Statement

The original contributions presented in the study are included in the article/supplementary material, further inquiries can be directed to the corresponding author/s.

## Ethics Statement

The animal study was reviewed and approved by the Department of Animal Science, Jiangxi University of Traditional Chinese Medicine.

## Author Contributions

CY conducted the experiments with assistance from WS, MZ, LW, RH, and MS. Experimental design, data analysis and interpretation, and writing were done by CY, WX, and YG. All authors contributed to the article and approved the submitted version.

## Conflict of Interest

The authors declare that the research was conducted in the absence of any commercial or financial relationships that could be construed as a potential conflict of interest.
